# Direct Carotid Artery Exposure for Acute Cerebral Infarction in Hybrid Angiography Suite: Indications and Limitations

**DOI:** 10.3389/fsurg.2021.819053

**Published:** 2022-01-27

**Authors:** Ching-Chang Chen, Chun-Ting Chen, Yi-Ming Wu, Po-Chuan Hsieh, Mun-Chun Yeap, Chien-Hung Chang, Chuan-Min Lin, Shao-Wei Chen

**Affiliations:** ^1^Department of Neurosurgery, Linkou Medical Center, Chang Gung Memorial Hospital, Chang Gung University, Taoyuan City, Taiwan; ^2^Division of Neuroradiology, Department of Radiology, Linkou Chang Gung Memorial Hospital & Chang Gung University, Taoyuan City, Taiwan; ^3^Department of Neurosurgery, New Taipei Municipal Tu-Cheng Hospital (Built and Operated by Chang Gung Medical Foundation), New Taipei City, Taiwan; ^4^Department of Neurology, Linkou Chang Gung Memorial Hospital & Chang Gung University, Taoyuan City, Taiwan; ^5^Department of Cardiovascular Surgery, Linkou Chang Gung Memorial Hospital, Chang Gung University, Taoyuan City, Taiwan

**Keywords:** endovascular treatment, carotid access, mechanical thrombectomy, carotid stent, carotid puncture

## Abstract

**Objectives:**

For the endovascular intervention of acute ischemic stroke, a transcervical route is an alternative approach in patients with challenging anatomical variations. Percutaneous puncture is a common way, but it can cause many fatal complications. Direct carotid artery exposure is an alternative for reducing complications. We demonstrate a technique of direct carotid exposure in patients for whom transfemoral or transbrachial approaches were impossible. We present patient outcomes and discuss the indications and limitations of this procedure.

**Methods:**

We retrospectively reviewed the cases of patients undergoing direct carotid exposure for acute ischemic stroke in a hybrid angiography suite and presented the details of the technique.

**Results:**

Among 548 consecutive patients with acute large vessel strokes who were treated by emergency endovascular thrombectomy or stenting between January 2015 and September 2020 in our center, only 8 (1.46%) required a transcervical approach. Of them, 7 underwent direct carotid exposure with successful recanalization and good clinical outcomes.

**Conclusions:**

Direct carotid exposure for endovascular stroke treatment is effective and advantageous in patients with challenging anatomical variations. Performing this procedure in carefully selected patients in the hybrid angiography suite can be beneficial in terms of open surgeries, saving time, and decreasing the risk of postoperative complications.

## Introduction

Endovascular intervention is effective for large vessel occlusion in patients with acute ischemic stroke (1–4). Rapid recanalization of the occluded vessels is pivotal for good clinical outcomes (5, 6). In most cases, the carotid artery can be accessed easily through the femoral artery approach. However, in patients with difficult anatomical variations, the procedure time can be prolonged and outcomes might be worse (5, 7–9). In these cases, the percutaneous carotid puncture is commonly employed (8, 10, 11). However, even successful recanalization, this procedure can cause fatal complications, such as pseudoaneurysm, dissection, and hematoma (6, 9, 10, 12), with the complication rate ranging from 14.3 to 36.8% (6, 10, 12) for acute mechanical thrombectomy in the literature.

Direct exposure of the carotid artery is an approach that can be considered in an emergency situation (13–15). Its advantages include the possibility of repeat punctures, easy achievement of hemostasis, and reduced bleeding under antiplatelet medications (14, 15). However, its true indications and techniques remain less discussed. Herein, we demonstrated the technique and outcomes of performing direct carotid exposure *via* the transcervical route; this technique can be used in cases of certain challenging anatomical variations, and closure can be performed safely in the hybrid angiography suite.

## Methods

### Patient Population and Selection

We retrospectively identified 548 consecutive patients with acute large vessel strokes who were treated with emergency endovascular thrombectomy or stent placement between January 2015 and September 2020 at our center. Patients with acute ischemic stroke admitted to our institution are treated by intravenous recombinant tissue plasminogen activator (rtPA) administration, endovascular thrombectomy, or both in accordance with the onset of symptoms and perfusion images. The individual treatment decision is an interdisciplinary process involving senior vascular neurologists and 4 experienced neurointerventionalists. Decision-making is based on clinical criteria (National Institute of Health Stroke Scale [NIHSS] score of >8 and <30) and the results of initial CT angiography (CTA) and perfusion CT (CTP). Endovascular intervention is considered in cases of occlusion of large arteries: the internal carotid artery (ICA) and M1 or M2 segments of the middle cerebral artery (MCA). Informed consent was obtained from all patients after informing them in detail about the risks, benefits, and alternatives of the procedures with multidisciplinary decision-making. The study was approved by the Institutional Review Board (IRB no. 201800342B0).

### Surgical Procedure of the Exposure and Closure of the Carotid Artery

Direct surgical dissection to expose the carotid artery was performed by senior vascular surgeons or neurosurgeons in a fully-equipped hybrid neuroendovascular operating room. After a 2 to 5 cm skin incision and dissecting along the anteromedial border of the sternocleidomastoid muscle, the carotid sheath was fully exposed. The common carotid arteries (CCAs) and ICAs were marked with vessel loops. An 18-gauge needle was then used to puncture the CCA, and a 6-Fr sheath was cannulated over a 0.035-inch guidewire (Terumo, Tokyo, Japan). Endovascular procedures were performed through this large-bore route. If retrograde contrast injection or retrograde stent placement is necessary, a second or even third puncture can be performed after closing the previous one. At the end of the endovascular intervention, the sheath was removed, the puncture sites were closed directly using Prolene sutures (Johnson & Johnson, Somerville, NJ, USA) or by a purse-string suture technique, and the skin was meticulously sutured without indwelling drain.

### Endovascular Procedures

The endovascular treatments of acute stroke in our institution were performed under general anesthesia by 4 experienced neurointerventionalists. After the 6-Fr sheath was inserted in the CCA, distal aspiration thrombectomy was performed if the occlusion site was accessible by an aspiration catheter (Sofia, Microvention, Tustin, USA; or ACE, Stryker, Fremont, USA). If aspiration did not achieve full recanalization, a stent retriever technique was used (Trevo, Stryker, Fremont, USA or ReVive, Codman, Raynham, USA). Balloon angioplasty was applied in case of severe residual stenosis; a stent was deployed if a dissecting wall was suspected. After the first puncture site was sutured, a second location was punctured if necessary, and the sheath was placed retrograde to the proximal CCA. Additional retrograde carotid stents (Wallstent, Boston Scientific, Natick, MA, USA or Precise, Cordis Endovascular, Miami, FL, USA) were then deployed until the proximal CCA or orifice of the brachiocephalic artery. Glycoprotein IIb/IIIa inhibitor (tirofiban) was administered immediately through the intra-arterial route and continuously with slow venous dripping if stents were placed during the procedure.

## Results

Among the 548 consecutive patients with acute large vessel strokes, the carotid artery could not be accessed through the transfemoral or transbrachial routes in 8 patients (1.46%); 7 patients (1.28%) required direct carotid artery exposure for endovascular mechanical thrombectomy or carotid stenting, and 1 required carotid puncture. The clinical data of all patients are summarized in Table 1. The initial NIHSS scores were 9–29. Recanalization (thrombolysis in cerebral infarction [TICI] ≥ 2b) was achieved in all patients (100%). For Patient 1, direct carotid puncture under echo guidance was performed. For Patient 6, the carotid artery was punctured at the aortic artificial graft in the chest cavity after type A aortic dissection surgery. For the remaining 6 patients, the carotid artery was exposed through surgical neck dissection. Two patients required adjuvant carotid stents for the dissecting vessels after aspiration. In three patients, the circulation was reestablished only through carotid stent deployment. Patient 1 had an ICA pseudoaneurysm at the puncture site; he died because of a large cerebral infarction. The other 7 patients had no procedure-related complications, and their symptoms improved during the follow-up period. At the 3-month follow-up, 6 of 7 patients had regained excellent neurological function (modified Rankin Scale [mRS] 0–2).

**Table 1:** Clinical data of the patients who undergo transcarotid approach for acute cerebral infarction.

**Case**	**Age/Gender**	**NIHSS (initial)**	**Etiology**	**Procedures**	**Result**	**3-month mRS**	**Complication**	**Time to change route**	**Time of Carotid exploration**	Time from puncture^*^to recanalization
Case 1	70 s/male	28	Type III arch	Direct puncture Aspiration Stent retrieve	TICI 2b	6	Pseudoaneurysm	65 min	min	>2 h
Case 2	60 s/male	9	Type III arch	Carotid expose Aspiration	TICI 3	0	No	15 min	22 min	75 min
Case 3	80 s/male	13	Type III arch	Carotid expose Aspiration	TICI 3	3	No	35 min	25 min	95 min
Case 4	40 s/male	20	Type A dissect ^#^	Carotid expose Aspiration Carotid stents	TICI 2b	1	No	0	17 min	123 min
Case 5	60 s/male	29	Type A dissect ^#^	Carotid expose Carotid stents	TICI 3	0	No	0	20 min	45 min
Case 6	60 s/male	19	Type A dissect ^#^	Graft puncture Carotid stents	TICI 3	1	No	0	0 min	35 min
Case 7	70 s/male	17	Type A dissect ^#^	Carotid expose Carotid stents	TICI 3	1	No	0	18 min	55 min
Case 8	60 s/male	18	Brachiocephalic Artery occlusion	Carotid expose Aspiration	TICI 3	1	No	25	22 min	92 min

* *Time since the first puncture from the first route for the endovascular procedure*.

# *Acute stroke occurred concurrently or immediately after type A dissection surgery*.

*mRS, modified Rankin scale; TICI, thrombolysis in cerebral infarction scale*.

### Indications and Case Illustrations

#### Indication 1: Tortuosity of the Aortic Arch and Carotid Artery

##### Patient 3. Type III Aortic Arch

**Figure 1:**
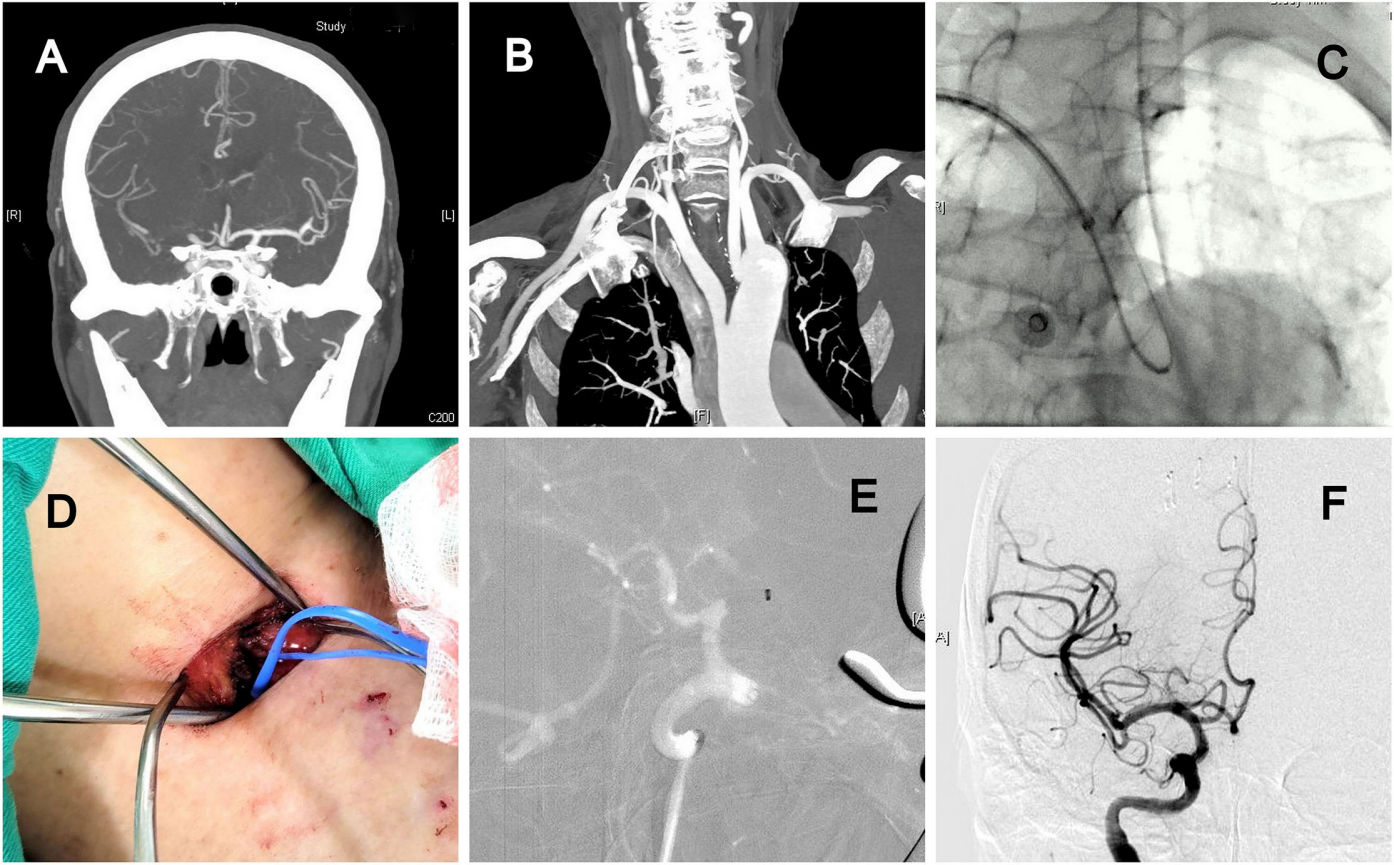
A patient had acute right middle cerebral artery (MCA) occlusion with a type III aortic arch and sharp angle between the right subclavian artery and right common carotid artery (CCA). **(A)** CT angiography (CTA) indicating total occlusion of the right MCA. **(B)** Type III aortic arch. **(C)** Sharp angle, which hindered the advancement of the long sheath to the carotid artery from the right hand. **(D)** Carotid artery exposure through a 2-cm skin incision. **(E)** Direct aspiration for mechanical thrombectomy. **(F)** Complete recanalization of the MCA.

A man in his 80s had a history of atrial fibrillation (AF), scoliosis, and spinal radiculopathy with lower limb weakness. He was found lying on the bathroom floor with left hemiplegia (NIHSS score: 13). The CTA revealed acute right MCA occlusion (Figure 1A) and a type III aortic arch (Figure 1B). Initially, an approach from the transfemoral and transbrachial routes was attempted, but a sharp angle between the right subclavian artery and CCA hindered the advancement of the long sheath to the carotid artery (Figure 1C). We then changed the patient position to facilitate a cervical carotid artery exposure through a small 2-cm skin incision (Figure 1D). After 2 passes of direct aspiration (Figure 1E), complete recanalization of the MCA territory was achieved (Figure 1F). The patient underwent 3-months of rehabilitation and medical control of AF, and he showed good recovery. The mRS was 3 because of residual left weakness and underlying spinal degeneration.

#### Indication 2: Type A Aortic Dissection With Innominate Artery Reimplantation

In patients who underwent aortic repair and innominate artery reimplantation after type A aortic dissection, approach for endovascular treatment was difficult, time-consuming, and hazardous. Four patients underwent the transcervical route because acute stroke developed immediately after emergency type A dissection surgery.

##### Patient 5. Acute Stroke Immediately After Type A Dissection Operation

**Figure 2:**
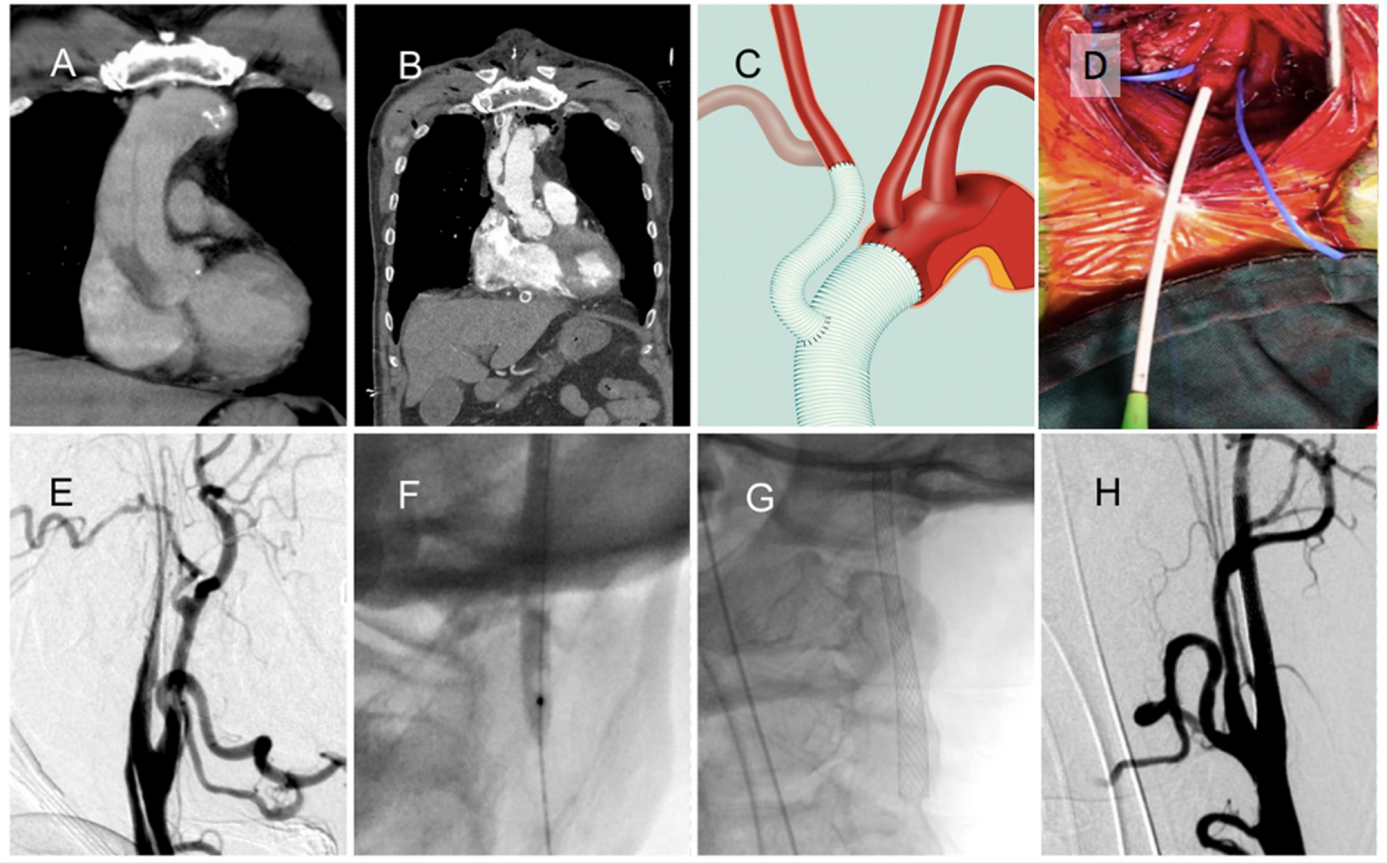
A patient with acute ischemic stroke after type A aortic dissection surgery. **(A)** Initial type A dissection before operation. **(B)** Ascending aortic arch replacement with graft placement, innominate artery debranching, and graft reimplantation. **(C)** Schematic of heart surgery. **(D)** Carotid exposure and puncture from the right neck. **(E)** Internal carotid artery (ICA) dissection and near-complete occlusion with string sign. **(F)** Balloon angioplasty of the occlusion site. **(G)** Carotid stent deployment. **(H)** Complete recanalization of the ICA.

A man in his 60s presented with sudden onset chest pain due to type A aortic dissection (Figure 2A). He underwent emergency ascending aortic arch replacement with graft, the innominate artery was debranched, and the innominate artery was reimplanted to the graft (Figures 2B,C). In the intensive care unit (ICU), he experienced seizures and left hemiplegia. The NIHSS score was 29, and the CTA revealed right ICA total occlusion. Carotid exploration was performed from the right neck (Figure 2D), and angiography revealed right ICA dissection and near-complete occlusion with string sign (Figure 2E). Balloon angioplasty (Figure 2F) and carotid stent deployment (Figure 2G) were applied. Complete recanalization was achieved within 1 h (Figure 2H). The patient regained consciousness and muscle power the next day. His mRS was almost 0, with no recurrence or seizure over the 3-month follow-up.

##### Patient 6. Direct Puncture From Artificial Graft of Type A Dissection After Open-Heart Surgery

**Figure 3:**
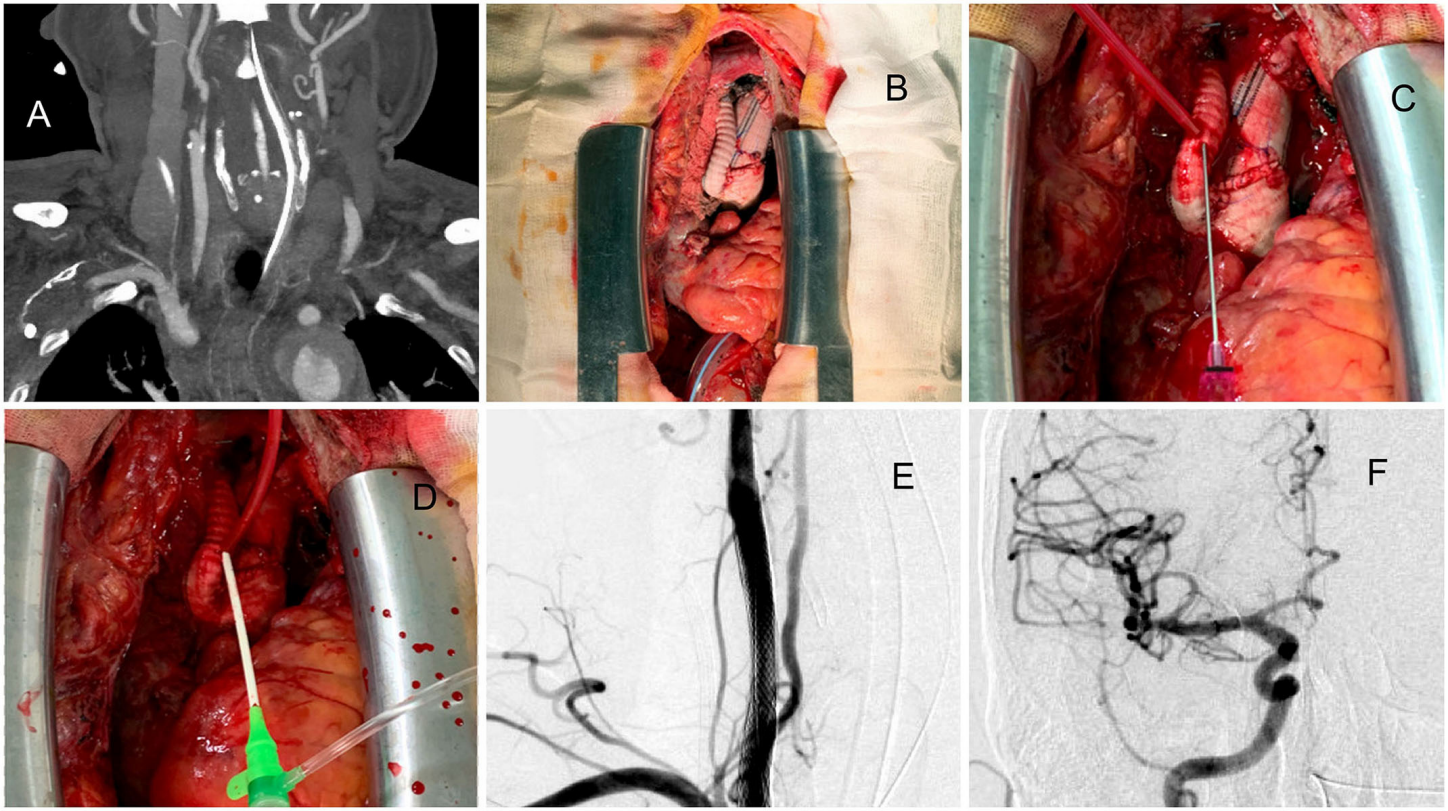
A case approached from exposure to the artificial aortic graft after type A aortic dissection surgery. **(A)** Dissecting extended to the right CCA and proximal ICA. **(B)** After open cardiac surgery, the reimplanted graft was exposed. **(C)** Direct puncture of the artificial graft. **(D)** The puncture needle was replaced with a 6-Fr sheath. **(E)** Two carotid stents were deployed to cover the proximal ICA to proximal CCA. **(F)** Thrombolysis in cerebral infarction (TICI) 3 results of the procedure.

A man in his 60s presented with sudden-onset chest tightness and left hemiparesis due to type A aortic dissection extending to the CCA and proximal ICA (Figure 3A). After cardiac surgery (ascending arch replacement and innominate artery reimplantation, Figure 3B), the neurosurgeon took over and directly punctured from the artificial graft in the chest cavity (Figure 3C) and then exchanged the puncture needle with a 6-Fr sheath (Figure 3D). Two carotid stents were deployed to cover the proximal ICA to proximal CCA (Figures 3E,F). The patient could raise his left hand 6 h after surgery. After 1-month of rehabilitation, his muscle power recovered and almost no neurologic deficits remained.

#### Indication 3: Anatomic Anomalies of the Aortic Arch or Brachiocephalic Trunk

##### Patient 8. Brachiocephalic Trunk Chronic Occlusion

**Figure 4:**
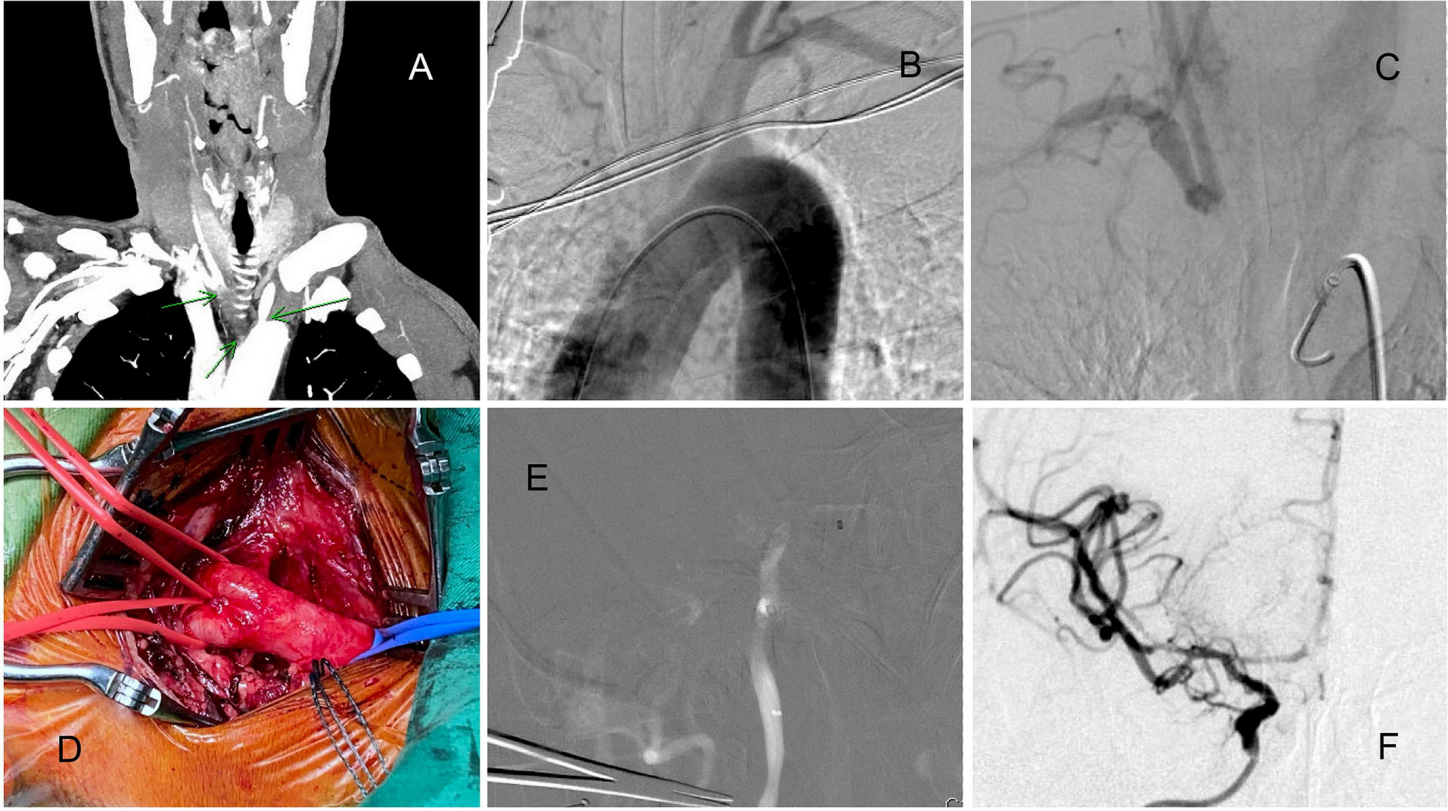
A patient with acute ICA and MCA total occlusion with chronic brachiocephalic trunk occlusion. **(A)** CTA indicating brachiocephalic trunk occlusion. **(B)** Aortogram indicating brachiocephalic trunk occlusion. **(C)** Retrograde collateral flow to the right CCA. **(D)** The carotid artery was directly explored from the neck. **(E)** The Sofia aspiration catheter was navigated to the occlusion site. **(F)** After 2 aspiration passes, total recanalization was achieved.

A man in his 60s presented with a 4-h history of sudden-onset left limb weakness (NIHSS score: 12). The CTA revealed right ICA and MCA occlusion (ASPECT score: 9), with the disappearance of the whole brachiocephalic trunk (Figure 4A). Initially, we attempted the routine femoral route. However, the aortogram indicated the total occlusion of the brachiocephalic trunk (Figure 4B) with slow retrograde collateral flow to the CCA (Figure 4C). Otherwise, the pulsation of the right upper limb was too weak to evaluate, and no obvious ischemic change of limbs and fingers was found. These findings implied chronic occlusion of the brachiocephalic trunk. Therefore, the carotid artery was directly explored from the neck (Figure 4D). The Sofia aspiration catheter was inserted from the carotid access (Figure 4E) for direct aspiration. After 2 aspiration passes, total recanalization with TICI 3 result was achieved (Figure 4F). Because of the initial attempts from the femoral artery and right arm, it took more time to approach. However, the patient's muscle power improved soon and near-complete recovery within 1-month.

## Discussions

It is the golden rule that the shorter time to achieve reperfusion, the better outcome to reach. Transfemoral arterial approach is the best route because of large diameter, smooth direction, and more familiarity by any neurointerventionalists. Transbrachial is smaller and large acute angle between subclavian and CCA. Patients who need transcervical approach must have some difficult anatomies, which let the interventionalists have no choice to do. Direct neck puncture is faster but may lead to more postoperative complications and is impossible to repeat punctures. In our series, open exposure might spend more time but still had a very good outcome.

Direct carotid exposure, or carotid cut down, is an alternative method for accessing the carotid artery in patients with challenging anatomical variations (8, 10, 11). The method provides a wide and clear field for performing repeated punctures and achieving hemostasis, and prevents fatal complications, such as pseudoaneurysm, dissection, and hematoma formation, by a direct puncture (6, 9, 10, 12). As an example, in our cohort, Patient 4 underwent 3 punctures and 5 stent deployments successfully without any complications (14). The indications of direct carotid artery exposure are as follows.

### Tortuosity of the Arch and Carotid Artery

**Figure 5:**
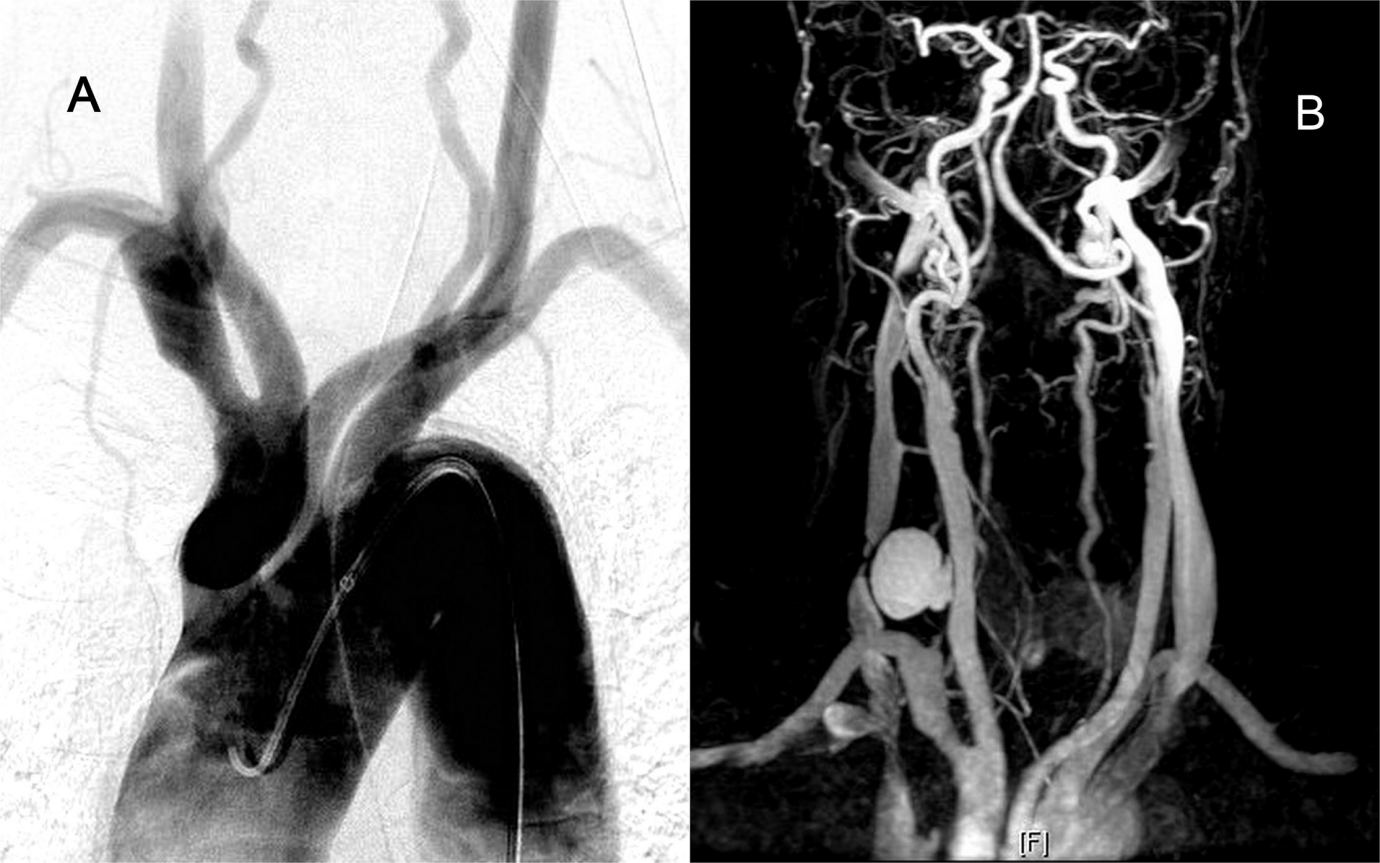
Patient 1 underwent a transcervical puncture. **(A)** The patient had a type III arch and a loop of the brachiocephalic trunk. **(B)** Postprocedural pseudoaneurysm formation.

Tortuous arch or carotid artery is a common indication for the transcervical approach. However, only 3 patients in our center required endovascular thrombectomy through the transcervical route. Patient 1 had a type III arch and a loop in the brachiocephalic trunk (Figure 5A). He underwent direct CCA puncture under ultrasound guidance after failure of the transfemoral and transbrachial routes. However, pseudoaneurysm formation (Figure 5B) was noted in follow-up images. Despite no aneurysm rupture and airway compression, the patient still died 2 weeks later because of large hemispheric infarction. After this experience, we never used direct neck puncture for patients with acute ischemic stroke having a tortuous arch. Percutaneous carotid puncture is often used but can cause fatal complications even after a successful procedure (6, 9, 10, 12). In these articles, the complication rate was ranged as high as from 14.3 to 36.8%. Because of the lack of soft tissue coverage and near the airway, achieving hemostasis at the percutaneous puncture site with compression or auto-suture devices is very difficult. Moreover, puncture site bleeding may occur due to previous rtPA administration, heparinization during the procedure, or use of antiplatelet medication after a stroke. Therefore, a percutaneous puncture may be prevented to routinely use. Many alternative techniques, such as stiff wire, double wire, Simon II catheters, and balloon anchor techniques (8), which we are familiar within our center, can help to overcome these anatomical challenges. In our series, only 3 out of 548 patients (0.5%) required access through transcervical routes, and 1 had complication by a direct puncture.

### Type A Aortic Dissection With Innominate Artery Reimplantation

Cardiac surgeries, especially aortic surgeries, have a high risk of postoperative stroke (16). In patients with type A aortic dissection, emergency aorta graft repair, stent coverage, and innominate artery reimplantation are common procedures. In these cases, access from transfemoral or transbrachial routes was nearly impossible and dangerous because of the following reasons: the steepness and the distance between the reimplanted innominate artery and the top of the arch (that becomes a large type III arch and superfluous loop, Figures 2B,C, 3B), residual dissection of the descending aorta, and difficulty of passing through the stent and graft. In most of these cases, recanalization can be achieved within 1 h after a carotid puncture, and excellent clinical results can be obtained. Patient 6 is the first reported case in the literature of the neuroendovascular procedure being performed directly through the artificial graft of the brachiocephalic trunk. After the procedure is complete, the puncture site can be directly sutured by a cardiovascular surgeon without any complications.

### Anatomic Anomalies of the Arch or Brachiocephalic Trunk

In patients with congenital aortic malformation, such as aortic coarctation (17), the transcervical route is indicated because the common approaches are impossible. In patients with chronic total occlusion or severe stenosis of the brachiocephalic trunk and left CCA orifice, the challenges encountered are poor support of the guiding catheter and difficulty in advancing the reperfusion catheter. In this situation, a transbrachial approach from the arms was another alternative. However, Patient 8 had chronic total occlusion of the brachiocephalic trunk and right limb pulsatile weakness, precluding a peripheral artery approach. This is also the first reported case of a carotid approach used because of brachiocephalic trunk occlusion.

Exposing the CCA in a fully-equipped hybrid neuroendovascular operation and the angiography room can assist the surgeon in carotid exposure and puncture, bypassing anatomically unfavorable areas and considerably shortening the time to successful revascularization (18). In addition, this technique has a low risk of post-closure bleeding and hematoma formation at the puncture site. Even if repeated punctures are required, direct sutures or purse-string sutures can be performed to avoid wound bleeding. A hybrid neuroendovascular suite allows a rapid transition between surgical and angiographic positions, reduces procedural time, and increases the chance of optimal patient outcomes (14, 15, 19). It also provides the option of using a transcervical approach through direct neck exposure without unnecessary or excessive repeated procedures in complicated cases, such as patients after open-heart surgery (Patient 7).

### Limitations

This study and procedure have some limitations. First, this study had a retrospective design with very few cases, limiting the generalizability of the results. The transcervical approach is not commonly used because the femoral route is accessible in most patients. Second, direct carotid exposure must be executed under general anesthesia, which inevitably takes more time than a percutaneous puncture. If carotid exposure is conducted after failure from other routes, factors, such as the administration of anesthesia and urgently calling a vascular surgeon further prolong the procedure time, potentially worsening the outcomes (as observed with Patients 1 and 3). Therefore, detailed interpretations of clinical and CTA data are crucial, and careful identification of these patients is paramount before beginning the interventional treatment. Notably, most experienced cardiovascular and neurosurgeons spend <20 min on carotid exploration using such a surgical approach. In most of our patients, these 20 min delays had no obvious effect on the final outcome. On the other hand, fortunately, in our series, there were no patients who had head and neck cancer with previous radiation. Indeed, in these patients, open surgery would be more difficult. Despite these limitations, our study demonstrated a reasonable time window with reliable results under rapid exposure and initial general anesthesia in the hybrid angiography suite.

## Conclusions

Direct surgical carotid exposure for endovascular stroke treatment is effective and has considerable advantages for patients with certain challenging anatomical variations. The approach in the hybrid angiography suite can facilitate surgical execution, save time, and decrease the risk of postoperative complications.

## Data Availability Statement

The raw data supporting the conclusions of this article will be made available by the authors, without undue reservation.

## Ethics Statement

The study was approved by the Institutional Review Board (no. 201800342B0). The patients/participants provided their written informed consent to participate in this study. Written informed consent was not obtained from the individual(s) for the publication of any potentially identifiable images or data included in this article.

## Author Contributions

C-CC: concept and design of the work and drafting the article. C-TC: data analysis and interpretation. Y-MW, P-CH, M-CY, C-HC, and C-ML: techniques review and conduct. S-WC: techniques consultation. All authors: final approval and data collection.

## Funding

The study was funded by Chang Gung Memorial Hospital [CMRPG3H0741 (C-CC)].

## Conflict of Interest

The authors declare that the research was conducted in the absence of any commercial or financial relationships that could be construed as a potential conflict of interest.

## Publisher's Note

All claims expressed in this article are solely those of the authors and do not necessarily represent those of their affiliated organizations, or those of the publisher, the editors and the reviewers. Any product that may be evaluated in this article, or claim that may be made by its manufacturer, is not guaranteed or endorsed by the publisher.
